# Gender and educational differences in work participation and working years lost in Norway

**DOI:** 10.5271/sjweh.4166

**Published:** 2024-09-01

**Authors:** Suzanne L Merkus, Rune Hoff, Rachel L Hasting, Karina Undem, Suzan JW Robroek, Jon Michael Gran, Ingrid Sivesind Mehlum

**Affiliations:** 1 Research Group for Work Psychology and Physiology, National Institute of Occupational Health, Oslo, Norway.; 2 Research Group for Occupational Medicine and Epidemiology, National Institute of Occupational Health, Oslo, Norway.; 3 Erasmus University Medical Center Rotterdam, Department of Public Health, PO Box 2040, 3000 CA Rotterdam, The Netherlands.; 4 Oslo Centre for Biostatistics and Epidemiology, Oslo University Hospital, Oslo, Norway.; 5 Oslo Centre for Biostatistics and Epidemiology, Department of Biostatistics, University of Oslo, Oslo, Norway.; 6 Department of Community Medicine and Global Health, Institute of Health and Society, University of Oslo, Oslo, Norway.

**Keywords:** disability pension, employment, retirement, sickness leave, working life, working life expectancy

## Abstract

**Objectives:**

This study aimed to quantify the duration of work participation and reasons for working years lost, according to gender and educational attainment, among a Norwegian population.

**Methods:**

Register data on labor market attachment between 2000–2015 were obtained from Statistics Norway. We included five cohorts: individuals turning 20 (N=323 333), 30 (N=386 006), 40 (N=388 962), 50 (N=358 745), and 60 years (N=284 425) between 1 January 2000 and 31 December 2005. Individuals were followed for ten years. Data completeness allowed calculation of the average time spent in work and years lost to health-related absences and non-employment states per cohort. Changes in state probabilities over time were also depicted. Mean differences between genders and educational levels, and corresponding 95% confidence intervals were based on 1000 bootstrap samples.

**Results:**

Both genders spent most time in work; however, per cohort, women worked approximately one year less than men. As cohorts aged, main reasons for working years lost changed from education and economic inactivity to sickness absence and disability pensioning; this trend was stronger for women than men. Individuals with a low education spent fewer years in work and more years in sickness absence and disability pensioning than highly educated peers. This difference tended to be larger for women and older cohorts.

**Conclusions:**

Per cohort, women participated one year less in work than men and, depending on age, spent more time in education, economic inactivity, sickness absence, and disability pensioning. Stronger educational gradients were seen for work and health-related absences for older cohorts and women.

To sustain viable social security systems, increasing work participation and reducing temporary and premature permanent withdrawal from work has been high on the political agenda of many western countries over the past decades. For example, pension reforms to increase retirement age have been seen across Europe as well as initiatives to reduce withdrawal from work due to health-related reductions in work capacity, ie, sickness absence and disability pensioning (1–4). However, it has been shown that the focus on reducing premature withdrawal from work does not necessarily lead to an increase in work participation. This is exemplified in Sweden, where new guidelines introduced in 2006 regarding the eligibility to receive a disability pension were followed by an increase in early retirement rather than work participation (5). It was hypothesized that older individuals with poor health, who were denied disability pension on medical grounds, may have reverted instead to early retirement to cope with their health issues (5). This shows the importance of assessing transitions between several work-related ‘states’, such as work, unemployment, sickness absence, disability pension and retirement, when aiming to understand factors that influence labor market attachment (6).

Several factors influence labor market attachment, including health status, and individual, demographic, work-related, and socioeconomic factors (5, 7–11). Although health-related reductions in work capacity may be reflected in several states, it is most directly reflected in time spent in longer-term sickness absence and disability pensioning as these states require a physician’s certification. Educational differences are one of the largest determinants of labor market attachment, with individuals with a low education spending 3–8 fewer years in work after the age of 50, and ≤10 years less after the age of 30 (8, 10). Gender differences in labor market attachment also exist, with women less likely to be in paid employment and more likely to be absent from work compared men (8–10). Norway is a country with a high number of older students and is known for gender equality, relatively small income differences, and a robust welfare system (12–14); therefore, it is a pertinent case for studying whether these characteristics translate into small educational and gender differences in labor market attachment.

Often, total time spent in various states throughout working life have been estimated using a period life-table approach, as is typically the case with working life expectancy (8, 10, 15, 16). This approach has been previously used to assesses working life expectancy of Norwegians >50 years of age (17). Such an approach uses a hypothetical cohort of individuals from many birth cohorts and predicts total average time spent in work and years lost during a life course as if it were one birth cohort (9–11, 18–20). While these estimates based on a period life-table approach are relevant for policy makers, a shortcoming is that this approach assumes that labor market circumstances, welfare policies, and health remain the same throughout the life course across birth cohorts. Yet, they are often presented as a prediction of the future expectations for a current working group (5, 19, 21, 22). A cohort life-table study provides a complementary approach (19). While timeliness can be a limitation, as such an approach requires completion of a cohort’s working life, it provides valuable insights into the work experiences of specific groups and is useful for capturing changes in norms and preferences among cohorts: from increased labor force participation of women to educational expansion and health improvement. This approach can complement that of Loichinger & Weber (17) in the Norwegian context.

This study aimed to quantify time spent in a spectrum of work-related states in a Norwegian population. The states represented work participation and reasons for working years lost. The latter includes unemployment, health-related reductions in work capacity (ie, ickness absence and disability pensioning), and non-employment with the potential for future work participation (education) or withdrawal from the labor market (economic inactivity, emigration, and retirement). Analyses were stratified according to gender and highest educational attainment. Understanding factors that influence labor market attachment is useful for policy makers to identify groups for whom interventions to increase work participation may be most beneficial.

## Methods

### Study population and design

This study extracted information from various national registries in Norway that are linked via the unique individual identification number. The source population was a cohort of working age (20–70 years) living in Norway between 2000 and 2010 (N=3 766 858); follow-up data was available until 2015. Our study population was restricted to individuals who lived in Norway on 1 January the year they turned 20, 30, 40, 50, or 60 years, respectively, between 2000 and 2005. Individuals entered the study on 1 January the year they were included and were followed for 10 years. Hence, we had five age-cohorts with a 10-year age range, ie, those aged 20–30 (born 1980–1985), 30–40 (born 1970–1975), 40–50 (born 1960–1965), 50–60 (born 1950–1955), and 60–70 (born 1940–1945) years. This meant that participants born in 1940, 1950, 1960, 1970, and 1980 were followed from 1 January 2000 until 31 December 2009, while participants born in 1941, 1951, 1961, 1971, 1981 were followed from 1 January 2001 until 31 December 2010, etc. Hence, the study period was from 1 January 2000 to 31 December 2014.

### Data sources

Access to the following databases for this study were obtained from Statistics Norway (SSB): the FD-Trygd events database on employment, welfare, demography, and income (23), and the National education database (24).

### Work participation and working years lost

We were able to distinguish between nine work-related states, ie, between individuals who were in work and the following eight reasons for working years lost: unemployment, sickness absence, disability pension, economic inactivity, (early) retirement, education, emigration, and death.

*Work* was defined as being registered as employed or receiving an income at some point during the year through self-employment; this state included parental and annual leave. *Unemployed* individuals were those registered as full-time job seekers. Registration was voluntary, and individuals were eligible to receive unemployment benefits for up to three years but could be registered as unemployed for longer (25, 26).

In Norway, *sickness absence* is granted if work capacity is—partially or fully—reduced due to illness or injury. The employer pays the salary for the first 16 calendar days of absence. From the 17^th^ day, benefits are paid by the Norwegian Labor and Welfare Administration; individuals are entitled to a maximum of 52 weeks of sickness absence. However, this period can be extended by up to five years with medical or vocational rehabilitation benefits (27). *Disability pension* is granted to individuals whose work capacity is permanently reduced by ≥40–50% due to illness or injury (26). It is possible to receive partial sickness absence benefits, medical or vocational rehabilitation benefits, or disability pension while simultaneously working partially. In this study, individuals were categorized in s*ickness absence* when they received sickness absence benefits or medical/vocational rehabilitation benefits for ≥50% of their contracted working hours, and they were categorized in *disability pension* when they received a disability pension due to ≥50% reduction in work capacity for a full-time position. For some medical/vocational rehabilitation benefits, we lacked information regarding grade, in those instances all episodes were categorized as receiving benefits for ≥50%.

*Education* was defined as enrolment in an educational institution along with a yearly income that was lower than twice the National Insurance scheme basic amount (28), which is the upper limit for being eligible for a full student loan (29). Individuals were considered as *emigrated* when they were no longer registered as a resident of Norway.

As our data spans from 2000–2015, it is worth mentioning that Norway underwent a pension reform in 2011 in which incentives to remain at work were introduced (30). However, large similarities between the old and new pension schemes include the right to draw a state pension from the statutory retirement age of 67 years, with the possibility to retire from the age of 62 (early retirement), given sufficient pension savings. Individuals may work and receive (early) retirement pension simultaneously; we defined *(early) retirement* as receiving a contractual early retirement pension (AFP) or state retirement pension ≥50% of the pensionable income.

*Death* was defined as being registered as having passed away. Individuals were considered *economically inactive* when they did not meet any of the criteria of the other states.

Most of the information used to categorize the states were updated daily. The datasets on *disability pension* and *(early) retirement* were updated monthly. For these datasets, we set the entry dates to the first of the month and exit dates to the last day of the month. Information on education was updated yearly, and we set the dates to follow the academic year, with entry into education on 1 August and exit on 31 July. Information on income was also updated yearly, for which we followed the calendar year from 1 January to 31 December.

### Gender and educational level

Gender was denoted as man/women. Educational level was highest attained educational level at the end of follow-up. Educational level was coded according to the Norwegian Standard Classification of Education (NUS2000) (31) into the following five levels: low (lower secondary or lower), low-intermediate (upper secondary, basic), intermediate (upper secondary, completed), intermediate-high (Bachelor level), and high (Master/PhD level).

### Data analysis

The primary study outcome was mean length of stay over 10 years in each work-related state. Previous studies estimating time spent in various states have used complex statistical methods, including multistate modelling, from which expected length of stay can be calculated (10, 32). Such analyses are suitable for datasets with missing data, eg, due to loss to follow-up (censoring) or lacking information on time spent in specific states. Such time to event data is truncated and requires methods to address these data issues. In contrast to previous studies, our data has 100% follow-up for the defined study periods and incorporates death and non-working states as competing risks. Therefore, after processing the data according to the steps described in the ‘State hierarchy’ section below, the time spent in each state was calculated as mean length of stay over 10 years in the corresponding state. The study’s secondary outcome was the change in state probabilities over the 10-year follow-up. These were estimated using gender specific cumulative transition rates between states, calculated by the Nelson-Aalen estimator, ie, the empirical transition matrix, which was then used with the Aalen-Johansen estimator to produce state probability curves for the 10-year follow-up period (33). Mean differences between genders and educational levels, and corresponding 95% confidence intervals (CI), were based on 1000 bootstrap samples.

R version 4.1.2 (RStudio version 1.4.1717, Boston, MA, USA) (34) was used together with the packages *data.table, mstate* and *timereg* to process and analyze the data.

As individuals are likely to have achieved their highest education by the age of 30, mean length of stay calculations were stratified by gender and highest attained educational level for the cohorts who turned 30, 40, 50, and 60 years. For individuals who turned 20 years, mean length of stay was estimated stratified by gender only.

### Data processing

To estimate mean length of stay in the various states, state histories during the 10-year follow-up were constructed for each individual. States must be mutually exclusive; therefore, when individuals could be classified simultaneously into more than one state, a state hierarchy was used to determine which states took precedence over others. For example, when individuals were registered as being in work as well as on sickness absence for ≥50%, sickness absence took precedence. In order of decreasing precedence, we prioritized (i) death, (ii) (early) retirement, (iii) disability pension, (iv) sickness absence, (v) education, (vi) employed (work state), (vii) unemployed, (viii) emigrated, (ix) self-employed (work state), and (x) economically inactive. Although the work state included both employed and self-employed individuals, self-employment was placed lower in the hierarchy as it was based on yearly income, which was less precise than the daily updated register data for employment. Individuals could move between work, unemployment, sickness absence, economic inactivity, and education (supplementary material, URL, figure S1). From these states it was possible to transition into disability pension, (early) retirement, and death. Transitions were also possible from disability pension to (early) retirement and death, and from (early) retirement to death. Death was defined as an absorbing state, ie, individuals could not transition out of the state.

## Results

**Figure 1:**
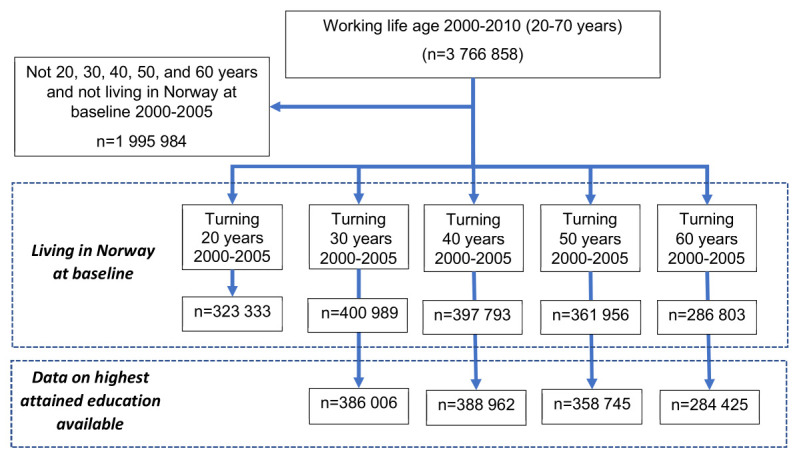
Flow chart of included individuals.

For the 20-year age cohort, we had information on those living in Norway at baseline, ie, on 1 January the year individuals turned 20 years between 2000–2005, for 323 333 individuals (figure 1). For the 30-year cohort, information on those living in Norway at baseline and their highest attained education was available for 386 006 individuals, for the 40-year cohort for 388 962, for the 50-year cohort for 358 745, and for the 60-year cohort for 284 425 individuals.

For each age cohort, a lower percentage of women than men were in work at baseline (supplementary table S1). At baseline, a higher percentage of women than men were receiving an education, economically inactive, or on sickness absence or disability pensioning.

### Gender differences in work participation and working years lost

**Figure 2:**
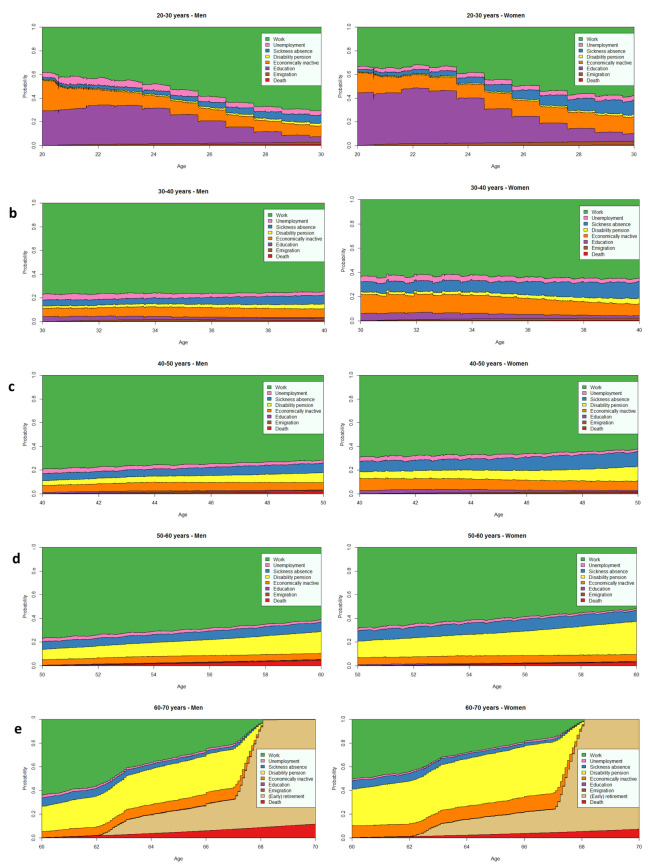
State probabilities throughout the 10-year follow-up for men and women in the cohorts a) 20-30 years, b) 30-40 years, c) 40-50 years, d) 50-60 years, and e) 60-70 years

Both men and women in each cohort spent most time in work (up to 7.64 years –table 1, figure 2). Gender differences seen at baseline continued into the 10-year follow-up (table 1, figure 2, and supplementary table S1 and S2). On average, women worked approximately one year shorter than men, this did not differ much across age cohorts. However, the main reasons why women spent more time out of work than men, changed as the cohorts aged. Differences in the 20-year cohort were mainly due to receiving an education (0.73 more years among women), whilst the largest differences were due to economic inactivity in the 30-year cohort (0.58 more years). Gender differences in the 40- to 60-year cohorts were mainly due to health-related absences from work, ie, compared to men, women spent more time in sickness absence in the 40-year cohort (0.38 more years), and in disability pensioning in the 50-year and 60-year cohorts (0.69 and 0.77 more years, respectively).

**Table 1:** The 10-year mean state durations of work participation, unemployment, sickness absence, disability pension, economic inactivity, and (early) retirement, stratified by age and gender. See Supplementary file S3 for state durations for education, emigration, and death. [SD=standard deviation; CI=confidence interval.]

		N	Work (years)				Unemployed (years)				Sickness absence (years)				Disability pension (years)				Economically inactive (years)				(Early) retirement (years)		
			Mean	SD	95% CI*		Mean	SD	95% CI*		Mean	SD	95% CI*		Mean	SD	95% CI*		Mean	SD	95% CI*		Mean	SD	95% CI*
20–30 years^a^																									
	Men	165 254	5.42	2.91			0.49	0.97			0.41	1.00			0.13	1.01			1.18	1.44					
	Women	158 079	4.42	2.53			0.37	0.79			0.65	1.20			0.11	0.92			1.29	1.53					
	Difference (men-women)*		1.01		0.99‒1.02		0.12		0.12‒0.13		-0.24		-0.25‒-0.23		0.02		0.02‒0.03		-0.12		-0.13‒-0.11				
30–40 years																									
	Men	195 790	7.64	3.00			0.38	0.92			0.57	1.22			0.26	1.48			0.75	1.48					
	Women	190 216	6.38	3.05			0.41	0.88			1.04	1.58			0.28	1.51			1.34	1.80					
	Difference (men-women)*		1.26		1.24‒1.28		-0.03		-0.04‒-0.03		-0.46		-0.47‒-0.45		-0.02		-0.03‒-0.01		-0.58		-0.59‒-0.57				
40–50 years																									
	Men	199 092	7.57	1.53			0.32	0.88			0.67	1.35			0.55	2.09			0.66	1.53					
	Women	189 870	6.67	3.51			0.32	0.82			1.05	1.74			0.78	2.47			0.87	1.79					
	Difference (men-women)*		0.90		0.88‒0.92		0.00		0.00‒0.01		-0.38		-0.39‒-0.37		-0.24		-0.25‒-0.22		-0.21		-0.22‒-0.20				
50–60 years																									
	Men	183 728	7.01	3.68			0.24	0.79			0.71	1.28			1.25	2.97			0.52	1.41					
	Women	175 017	6.07	1.65			0.21	0.69			0.95	1.53			1.94	3.59			0.61	1.65					
	Difference (men-women)*		0.93		0.91‒0.96		0.04		0.03‒0.04		-0.24		-0.25‒-0.23		-0.69		-0.71‒-0.67		-0.09		-0.10‒-0.08				
60–70 years																									
	Men	143 125	3.12	2.84			0.13	0.66			0.35	0.65			2.13	3.09			0.63	1.63			3.09	2.01	
	Women	141 300	2.40	2.75			0.10	0.60			0.34	0.65			2.91	3.39			0.88	2.11			3.02	1.80	
	Difference (men-women)*		0.72		0.70‒0.74		0.03		0.02‒0.03		0.01		0.01‒0.02		-0.77		-0.80‒-0.75		-0.25		-0.26‒-0.24		0.06		0.05‒0.08

^a^ The 20-year age cohort is the only cohort that included individuals for whom educational information was not available. *Mean difference between genders and 95% CI based on 1000 bootstrapped samples.

### Educational differences in work participation and working years lost

Educational gradients were seen for time spent in several states and for most of the age cohorts (tables 2 & 3, supplementary tables S3–S6b). The clearest trend was seen for work and disability pension. Compared to individuals with a high education, individuals with a low education spent less time in work (men up to 3.32 years; women up to 3.59 years) and more time on disability pension (men up to 2.75 years; women up to 3.13 years). The educational gradients were strongest for the older age cohorts, ie, 50- and 60-year-olds, and tended to be slightly larger for women than men.

**Table 2a:** The 10-year mean state durations of work participation, unemployment, sickness absence, disability pension, economic inactivity, and (early) retirement for men, per age cohort, stratified by educational level. See Supplementary file S4 for education, emigration, and death.

Men		N	%	Work (years)				Unemployed (years)				Sickness absence (years)				Disability pension (years)				Economically inactive (years)		
				Mean	SD	95% CI*		Mean	SD	95% CI*		Mean	SD	95% CI*		Mean	SD	95% CI*		Mean	SD	95% CI*
30–40 years		195 790																				
	Low education	31 044	16	5.55	3.75			0.83	1.36			1.12	1.72			1.08	2.94			1.07	1.77	
	Low-intermediate	11 568	6	7.00	3.28			0.59	1.15			0.90	1.55			0.42	1.83			0.82	1.51	
	Intermediate	81 951	42	8.07	2.64			0.33	0.84			0.58	1.17			0.12	0.97			0.68	1.43	
	Intermediate-high	47 858	24	8.11	2.57			0.25	0.68			0.34	0.88			0.05	0.60			0.70	1.40	
	High education	23 369	12	8.24	2.43			0.15	0.50			0.15	0.57			0.01	0.32			0.66	1.28	
	Diff (high-low)*			2.68		2.63‒2.74		-0.68		-0.69‒-0.66		-0.97		-0.99‒-0.95		-1.07		-1.10‒-1.03		-0.41		-0.44‒-0.39
40–50 years		199 092																				
	Low education	47 522	24	6.09	1.62			0.52	1.13			1.04	1.68			1.35	3.16			0.76	1.62	
	Low-intermediate	13 450	7	7.29	1.51			0.36	0.92			0.79	1.47			0.71	2.37			0.65	1.51	
	Intermediate	80 562	40	8.03	1.46			0.26	0.78			0.64	1.27			0.32	1.58			0.59	1.46	
	Intermediate-high	38 600	19	8.08	1.53			0.26	0.77			0.45	1.10			0.20	1.25			0.67	1.53	
	High education	18 958	10	8.50	1.56			0.15	0.60			0.21	0.71			0.07	0.73			0.68	1.56	
	Diff (high-low)*			2.41		2.36‒2.46		-0.37		-0.39‒-0.36		-0.83		-0.84‒-0.81		-1.28		-1.31‒-1.25		-0.08		-0.11‒-0.06
50–60 years		183 728																				
	Low education	36 029	19	5.13	4.15			0.35	0.94			0.96	1.50			2.66	3.98			0.49	0.65	
	Low-intermediate	46 046	25	6.77	3.76			0.25	0.79			0.80	1.34			1.45	3.16			0.46	0.57	
	Intermediate	49 110	27	7.45	3.35			0.23	0.74			0.74	1.27			0.87	2.47			0.48	1.34	
	Intermediate-high	34 563	19	7.88	3.15			0.20	0.75			0.50	1.09			0.55	2.03			0.61	1.58	
	High education	17 980	10	8.45	2.67			0.11	0.55			0.29	0.81			0.23	1.31			0.66	3.15	
	Diff (high-low)*			3.32		3.27‒3.38		-0.24		-0.26‒-0.23		-0.67		-0.69‒-0.65		-2.43		-2.48‒-2.39		0.17		0.14‒0.20
60–70 years		143 125																				
	Low education	36 819	26	2.12	2.58			0.13	0.65			0.38	0.68			3.33	3.39			0.40	1.33	
	Low-intermediate	39 887	28	2.96	2.76			0.13	0.67			0.39	0.69			2.28	3.14			0.59	1.59	
	Intermediate	31 154	22	3.07	2.72			0.14	0.70			0.36	0.66			1.82	2.94			0.89	1.96	
	Intermediate-high	22 295	15	3.93	2.75			0.13	0.66			0.32	0.60			1.24	2.50			0.65	1.59	
	High education	12 970	9	5.19	2.74			0.08	0.53			0.23	0.51			0.58	1.79			0.72	1.68	
	Diff (high-low)*			3.07		3.02‒3.12		-0.05		-0.06‒-0.03		-0.15		-0.16‒-0.14		-2.75		-2.79‒-2.70		0.32		0.29‒0.35

* Mean difference between high and low education and 95% CI based on 1000 bootstrapped samples.

**Table 2b:** The 10-year mean state durations of work participation, unemployment, sickness absence, disability pension, economic inactivity, and (early) retirement for men, per age cohort, stratified by educational level. See Supplementary file S4 for education, emigration, and death.

		N	%	(Early) retirement (years)		
				Mean	SD	95% CI*
60–70 years		143 125				
	Low education	36 819	26	2.89	2.01	
	Low-intermediate	39 887	28	3.11	2.01	
	Intermediate	31 154	22	3.21	2.07	
	Intermediate-high	22 295	15	3.34	2.04	
	High education	12 970	9	2.87	1.74	
	Diff (high-low)*			-0.03		-0.06‒0.01

* Mean difference between high and low education and 95% CI based on 1000 bootstrapped samples.

**Table 3a:** The 10-year mean state durations of work participation, unemployment, sickness absence, disability pension, economic inactivity, and (early) retirement for women, per age cohort, stratified by educational level. See Supplementary file S4 for education, emigration, and death.

Women		N	%	Work (years)				Unemployed (years)				Sickness absence (years)				Disability pension (years)				Economically inactive (years)		
				Mean	SD	95% CI*		Mean	SD	95% CI*		Mean	SD	95% CI*		Mean	SD	95% CI*		Mean	SD	95% CI*
30–40 years		190 216																				
	Low education	24 784	13	4.14	3.49			0.85	1.27			1.67	2.15			1.24	3.09			1.75	2.29	
	Low-intermediate	10 648	6	5.76	3.32			0.56	1.05			1.41	1.94			0.53	2.07			1.48	2.06	
	Intermediate	57 636	30	6.53	2.98			0.47	0.92			1.13	1.63			0.19	1.20			1.36	1.86	
	Intermediate-high	74 204	39	6.88	2.67			0.26	0.64			0.85	1.32			0.07	0.70			1.22	1.60	
	High education	22 944	12	7.09	2.53			0.21	0.55			0.53	0.94			0.02	0.37			1.13	1.42	
	Diff (high-low) *			2.94		2.89‒3.00		-0.64		-0.66‒-0.62		-1.14		-1.17‒-1.11		-1.22		-1.26‒-1.18		-0.62		-0.66‒-0.59
40–50 years		189 870																				
	Low education	44 838	24	5.12	3.94			0.50	1.06			1.41	2.07			1.78	3.54			0.99	1.96	
	Low-intermediate	16 709	9	6.52	3.61			0.30	0.81			1.20	1.86			0.96	2.70			0.88	1.86	
	Intermediate	57 799	30	7.04	3.29			0.31	0.80			1.03	1.68			0.55	2.06			0.87	1.80	
	Intermediate-high	56 249	30	7.30	3.09			0.21	0.64			0.86	1.51			0.33	1.61			0.79	1.68	
	High education	14 275	7	7.80	2.80			0.18	0.59			0.55	1.19			0.15	1.10			0.75	1.53	
	Diff (high-low) *			2.69		2.63‒2.74		-0.32		-0.34‒-0.31		-0.86		-0.89‒-0.84		-1.63		-1.67‒-1.59		-0.24		-0.27‒-0.21
50–60 years		175 017																				
	Low education	37 361	21	4.11	1.74			0.29	0.82			1.07	1.67			3.61	4.35			0.65	1.74	
	Low-intermediate	55 799	32	5.90	1.59			0.21	0.68			0.97	1.54			2.18	3.75			0.57	1.59	
	Intermediate	30 585	17	6.71	1.68			0.22	0.71			1.01	1.53			1.24	2.91			0.65	1.68	
	Intermediate-high	43 086	25	7.23	1.62			0.14	0.57			0.85	1.41			0.94	2.62			0.59	1.62	
	High education	8 186	5	7.69	1.64			0.15	0.63			0.62	1.20			0.48	1.91			0.68	1.64	
	Diff (high-low) *			3.59		3.51‒3.66		-0.14		-0.15‒-0.12		-0.44		-0.47‒-0.42		-3.13		-3.19‒-3.07		0.02		-0.02‒0.06
	60–70 years	141 300																				
	Low education	44 228	31	1.53	2.38			0.09	0.53			0.29	0.62			3.90	3.49			0.98	2.31	
	Low-intermediate	56 195	40	2.43	2.73			0.13	0.69			0.35	0.65			2.83	3.37			0.90	2.13	
	Intermediate	13 733	10	3.05	2.88			0.13	0.66			0.39	0.70			2.13	3.11			0.93	2.12	
	Intermediate-high	23 614	17	3.29	2.74			0.07	0.45			0.39	0.66			1.98	3.01			0.64	1.64	
	High education	3 530	2	4.61	2.92			0.08	0.50			0.38	0.68			1.09	2.40			0.61	1.57	
	Diff (high-low) *			3.08		2.98‒3.17		-0.01		-0.02‒0.01		0.09		0.06‒0.11		-2.80		-2.89‒-2.71		-0.37		-0.42‒-0.31

*Mean difference between high and low education and 95% CI based on 1000 bootstrapped samples.

**Table 3b:** The 10-year mean state durations of work participation, unemployment, sickness absence, disability pension, economic inactivity, and (early) retirement for women, per age cohort, stratified by educational level. See Supplementary file S4 for education, emigration, and death.

		N	%	(Early) retirement (years)		
				Mean	SD	95% CI*
60–70 years		141 300				
	Low education	44 228	31	2.78	1.64	
	Low-intermediate	56 195	40	3.06	1.81	
	Intermediate	13 733	10	3.07	1.82	
	Intermediate-high	23 614	17	3.39	1.97	
	High education	3 530	2	2.94	1.76	
	Diff (high-low) *			0.17		0.10-0.23

*Mean difference between high and low education and 95% CI based on 1000 bootstrapped samples.

Albeit less pronounced than for work and disability pensioning, the 30-year to 50-year cohorts additionally showed educational gradients in unemployment and sickness absence (tables 2 & 3, supplementary tables S4–S6b). The largest differences were seen in the 30-year age cohort: low educated women spent 1.14 more years in sickness absence and 0.64 more years on unemployment than their high educated peers. For men the corresponding differences were 0.97 and 0.68 years, respectively.

## Discussion

### Summary of main findings

In general, the cohorts spent most time in work throughout the 10-year follow-up period; this was also the case for the 60-year cohort until statutory retirement at age 67. Women spent approximately one year shorter in work per decade than men, this did not change substantially as the cohorts aged. As the cohorts aged, reasons for most working years lost changed from receiving an education and being economically inactive, to sickness absence and disability pensioning. Women tended to spend more time in these states than men. Educational gradients were seen for work and health-related absences (sickness absence and disability pensioning), where those with a high education spent more years in work and fewer years in health-related absences than those with a low education. The educational gradients for work and disability pensioning were slightly increased for the older age cohorts and tended to be larger for women than men.

### Strengths and limitations

The main strength of the study is the use of register data. These data are not subject to recall bias and allowed us to follow large samples of individuals over a long period without the risk of drop-out. The findings are generalizable to similar populations, but caution should be taken when generalizing to immigrant populations as a large proportion of this group missed information on education. Additionally, due to cohort effects, caution is warranted when generalizing to similar age groups in later years. The register data were detailed and complete: they described most states at a daily level and provided information on all possible work-related states. A weakness of register data is the lack of information on factors such as health status and direct assessments of work exposures that are relevant to understanding the dynamics to labor market attachment. Some states were less accurate, which may have led to misclassification, eg, for self-employment and education, as daily updated information was not available, and time spent in these states may be overestimated. Additionally, registering as a job seeker is voluntary, so not all may register themselves as unemployed, which may underestimate time spent in unemployment. To minimize inaccuracies, we took the following steps during data processing: (i) we differentiated between individuals obtaining an education with and without an income that was compatible with being a student, and (ii) when developing the state hierarchy, states based on the least detailed or accurate data sources, ie, self-employment and emigration, were placed at the lower end of the hierarchy. Also, the fact that some data on medical/vocational rehabilitation lacked the grade of absence may have led to misclassification. However, we do not expect this to have influenced our results to a large extent as the data sources that had information on grade of these absences suggest that those with <50% absence made up a small percentage of our data. Lastly, the reader should keep in mind that state durations relate to our chosen definition of spending ≥50% of the time or receiving an equivalent of ≥50% in benefits or pension, in the respective states. This means that time spent in work was underestimated. For example, figure 2 shows that almost all individuals >68 years were allocated to the (early) retirement state because they almost all received ≥50% state pension, even though some were also (partially) in work.

### Interpretation of the findings

Like other European countries, duration of work participation in Norway was shorter for women than men (7, 8, 10). The gender differences in the present study were already present at the start of follow-up and could largely be explained by women spending more time than men in education—which can be seen as an investment in future work participation—but also in economic inactivity, sickness absence, and disability pensioning. This is similar to The Netherlands, although the difference in economic inactivity was less pronounced in Norway (10). This could potentially be explained by women in Norway being more encouraged and expected to participate in work, facilitated by, eg, long maternity leave and good daycare systems (35). Across Europe, similar to the differences found in Norway, women tend to experience more sickness absence and disability pensioning than men (10, 36, 37). This could suggest that this gender gap is less dependent on differences in culture and social security systems than, for example, the gender gap in economic inactivity.

The gender gap in health-related absence from work has previously been attributed to more women than men having musculoskeletal or psychological-related diagnoses (11, 36), which is also the case in Norway (38). Reasons for this remain unknown. Possible explanations may include biological differences between the sexes (39). Poor work-life balance has shown to increase the risk of sickness absence (40), and with women performing the majority of household chores this double burden could be another explanation for the gender gap (41). Alternatively, work demands in female-dominated occupations, e.g., in healthcare, where job tasks can be both physically and emotionally demanding, may increase the risk of musculoskeletal and psychological complaints (39). All-in-all, although Norway is considered one of the top ranked countries regarding gender equality in labor force participation (12),our findings highlight the need for continued efforts to reduce the gender gap in work participation and health-related reasons for working years lost.

Like other European countries, duration of work participation in Norway was shorter for individuals with a lower versus higher education (7, 8, 10, 19). The reasons for shorter work participation were, like in The Netherlands, attributed to lower educated men and women in Norway spending more time in unemployment and work disability (10). However, the large educational gradient for economic inactivity among women in The Netherlands was not seen in Norway (10). Absence due to poor health for both men and women was high in the youngest cohort, which could indicate that young individuals with a disability struggled to enter or remain in the labor market. These individuals likely have a capacity for work that is not always utilized. Our findings suggest that efforts to increase work participation in Norway may be most beneficial for individuals with a lower education by addressing causes of unemployment, sickness absence and disability pensioning.

Including various work-related states in our analyses has shown that the main reasons for exit from work after the age of 30 in Norway were related to poor health and that there were large educational differences in time spent in health-related states. Additionally, a shift from sickness absence to disability pensioning as the main reason for working years lost as the cohorts aged—indicating a shift from temporary to permanent exit from work—was most pronounced among individuals with low educational attainment. This pattern may be explained by two mechanisms related to educational differences. First, lower educated individuals have a higher risk for poor health, for example through lifestyle choices (42). Second, lower educated individuals are more likely to end up in manual occupations that have strenuous work demands. Therefore, the shift from sickness absence to disability may reflect the cumulative effect of educational differences in health as well as exposure to adverse working conditions (19, 43). Previous sick leave and lower educational attainment increase the risk for disability pensioning (44–47), suggesting that sick leave and lower educational attainment may start a process of marginalization from the labor market (44). Addressing sickness absence and increasing work participation early in the working life-course, especially for women, may reduce the risk of disability pensioning at a later age.

### Future directions

We encourage future studies to consider a life-course perspective of work participation to account for the dynamics between work participation and exit from work. We further encourage the simultaneous investigation of several routes by which individuals can exit work. This increases our understanding of reasons for non-participation, provides insight for policy makers to target the states of withdrawal that require most attention at given ages, and provides insight into the effectiveness of policy changes when evaluating such interventions.

### Concluding remarks

Independent of age cohort, most time was spent in work, with women participating approximately a year less in work than men. As the cohorts aged, reasons for most working years lost changed from receiving an education and being economically inactive to sickness absence and disability pensioning; women spent more time in these states than men. Additionally, clear educational gradients were seen for work, where lower educated individuals spent less time in work than their high educated peers. This gradient could largely be explained by lower educated individuals spending more time in health-related absences. These gradients were more pronounced for women and older age cohorts. Thus, at a population level, women and individuals with a lower education might be in most need of interventions to increase work participation. Such interventions should consider targeting individuals early in working life to impede a process of marginalization from the labor market.

## Supplementary material

Supplementary material
