# Supplementary material for: Gender and educational differences in work participation and working years lost in Norway
Source: Scand J Work Environ Health. 2024 Aug 30;50(6):426–36. doi: 10.5271/sjweh.4166 (PMC11391266; doi:10.5271/sjweh.4166)
Supplement: Supplementary material [file SJWEH-50-426-S001.pdf]

Gender and educational differences in work participation and working years lost in Norway[1]

by Suzanne L Merkus, PhD,[2] Rune Hoff, PhD, Rachel L Hasting, MPhil, Karina Undem, MPhil, Suzan JW Robroek, PhD, Jon Michael Gran, PhD, Ingrid Sivesind Mehlum, PhD

1. Supplementary material
2. Correspondence to: Suzanne Merkus, National Institute of Occupational Health, Pb 5330 Majorstuen, 0304 Oslo, Norway. [E-mail: suzanne.merkus@stami.no]

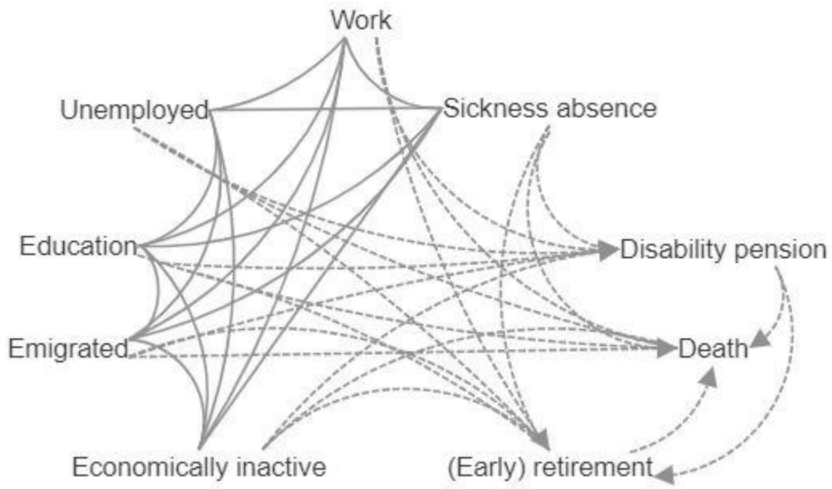

Figure S1. Illustration of possible transitions between the work-related states. Solid lines connect states for which mutual transitions were possible. Dashed lines with arrow heads connect states for which transitions were only possible in one direction.

Table S1. Labour market attachment at baseline for the cohorts who turned 20, 30, 40, 50, and 60 (1st January 2000-2005), stratified by gender

	n	Work %	Unemployed %	Sickness absence %	Disability pension %	Economically inactive %	Education %
**20 years[a]**							
Men	165 254	39.2	3.1	1.7	0.7	26.1	29.3
Women	158 079	33.9	2.0	1.9	0.6	17.1	44.6
**30 years**							
Men	195 790	77.6	4.5	4.8	1.9	7.1	4.2
Women	190 216	64.1	4.2	7.9	1.9	15.8	6.1
**40 years**							
Men	199 092	79.8	3.6	5.7	4.1	5.8	1.1
Women	189 870	69.7	3.4	8.4	5.7	10.1	2.6
**50 years**							
Men	183 728	77.4	2.7	6.2	8.8	4.4	0.5
Women	175 017	68.9	2.2	8.3	13.9	5.7	0.9
**60 years**							
Men	143 125	64.3	2.2	7.0	21.4	4.9	0.1
Women	141 300	50.7	1.5	6.8	31.1	9.9	0.1

[a] *The 20-year age cohort is the only cohort that included individuals for whom educational information was not available.*

Table S2. The 10-year mean state duration in education, emigration, and death, per age cohort, stratified by gender

		Education (years)			Emigration (years)			Death (years)		
	**n**	**m**	**(sd)**	**95% CI***	**m**	**(sd)**	**95% CI***	**m**	**(sd)**	**95% CI***
**20-30 years**[a]										
Men	165 254	2.22	(2.38)		0.11	(0.82)		0.05	(0.56)	
Women	158 079	2.95	(2.41)		0.20	(1.15)		0.02	(0.34)	
Diff (men-women)*		-0.73		-0.75–-0.71	-0.09		-0.10–-0.09	0.03		0.03–0.03
**30-40 years**										
Men	195 790	0.22	(0.92)		0.12	(0.88)		0.05	(0.57)	
Women	190 216	0.40	(0.98)		0.14	(0.95)		0.02	(0.36)	
Diff (men-women)*		-0.18		-0.18–-0.17	-0.02		-0.02–-0.01	0.03		0.03–0.03
**40-50 years**										
Men	199 092	0.08	(0.45)		0.08	(0.73)		0.08	(0.70)	
Women	189 870	0.20	(0.70)		0.06	(0.65)		0.05	(0.56)	
Diff (men-women)*		-0.12		-0.12–-0.11	0.02		0.01–0.02	0.03		0.02–0.03
**50-60 years**										
Men	183 728	0.03	(0.26)		0.04	(0.52)		0.20	(0.90)	
Women	175 017	0.05	(0.35)		0.03	(0.47)		0.13	(1.10)	
Diff (men-women)*		-0.02		-0.03–-0.02	0.01		0.01–0.01	0.07		0.06–0.08
**60-70 years**										
Men	143 125	0.01	(0.10)		0.03	(0.39)		0.52	(1.72)	
Women	141 300	0.00	(0.10)		0.02	(0.33)		0.32	(1.38)	
Diff (men-women)*		0.00		0.00–0.00	0.01		0.01–0.01	0.20		0.19–0.21

[a] *The 20-year age cohort is the only cohort that included individuals for whom educational information was not available.*
**Mean difference between genders and 95% CI based on 1000 bootstrapped samples*

Table S3. The 10-year mean state durations in education, emigration, and death, for men, per age cohort, stratified by educational level

			Education (years)			Emigration (years)			Death (years)		
	n	(%)	m	(sd)	95% CI*	m	(sd)	95% CI*	m	(sd)	95% CI*
**30-40 years**	195 790										
Low education	31 044	(16)	0.09	(0.39)		0.12	(0.89)		0.13	(0.93)	
Low-intermediate	11 568	(6)	0.08	(0.35)		0.12	(0.89)		0.08	(0.74)	
Intermediate	81 951	(42)	0.10	(0.45)		0.07	(0.68)		0.04	(0.51)	
Intermediate-high	47 858	(24)	0.40	(1.01)		0.15	(0.97)		0.02	(0.38)	
High education	23 369	(12)	0.53	(1.19)		0.25	(1.21)		0.01	(0.31)	
Diff (high-low)*			0.44		0.42–0.46	0.13		0.11–0.14	-0.12		-0.13–-0.10
**40-50 years**	199 092										
Low education	47 522	(24)	0.04	(0.24)		0.06	(0.62)		0.15	(0.97)	
Low-intermediate	13 450	(7)	0.03	(0.23)		0.08	(0.69)		0.09	(0.79)	
Intermediate	80 562	(40)	0.05	(0.30)		0.05	(0.55)		0.06	(0.60)	
Intermediate-high	38 600	(19)	0.19	(0.71)		0.12	(0.88)		0.04	(0.52)	
High education	18 958	(10)	0.16	(0.70)		0.20	(1.15)		0.04	(0.49)	
Diff (high-low)*			0.12		0.11–0.13	0.14		0.12–0.16	-0.11		-0.12–-0.10
**50-60 years**	183 728										
Low education	36 029	(19)	0.01	(0.14)		0.04	(0.50)		0.35	(1.45)	
Low-intermediate	46 046	(25)	0.01	(0.13)		0.02	(0.38)		0.22	(1.16)	
Intermediate	49 110	(27)	0.02	(0.19)		0.03	(0.44)		0.16	(1.00)	
Intermediate-high	34 563	(19)	0.07	(0.41)		0.06	(0.62)		0.12	(0.85)	
High education	17 980	(10)	0.06	(0.44)		0.10	(0.80)		0.10	(0.78)	
Diff (high-low)*			0.05		0.04–0.06	0.06		0.05–0.07	-0.25		-0.27–-0.23
**60-70 years**	143 125										
Low education	36 819	(26)	0.00	(0.05)		0.02	(0.37)		0.73	(2.01)	
Low-intermediate	39 887	(28)	0.00	(0.05)		0.02	(0.30)		0.52	(1.73)	
Intermediate	31 154	(22)	0.00	(0.09)		0.03	(0.43)		0.47	(1.66)	
Intermediate-high	22 295	(15)	0.01	(0.15)		0.03	(0.45)		0.35	(1.45)	
High education	12 970	(9)	0.02	(0.20)		0.04	(0.49)		0.28	(1.27)	
Diff (high-low)*			0.01		0.01–0.02	0.02		0.01–0.03	-0.45		-0.48–-0.42

**Mean difference between genders and 95% CI based on 1000 bootstrapped samples*

Table S4. The 10-year mean state durations in education, emigration, and death, for women, per age cohort, stratified by educational level

			Education (years)			Emigration (years)			Death (years)		
	n	(%)	m	(sd)	95% CI*	m	(sd)	95% CI*	m	(sd)	95% CI*
**30-40 years**	190 216										
Low education	24 784	(13)	0.15	(0.50)		0.14	(0.96)		0.05	(0.59)	
Low-intermediate	10 648	(6)	0.10	(0.41)		0.13	(0.94)		0.03	(0.40)	
Intermediate	57 636	(30)	0.20	(0.61)		0.09	(077)		0.02	(0.35)	
Intermediate-high	74 204	(39)	0.57	(1.16)		0.14	(0.98)		0.01	(0.28)	
High education	22 944	(12)	0.77	(1.39)		0.25	(1.23)		0.01	(0.22)	
Diff (high-low)*			0.62		0.60–0.64	0.11		0.09–0.13	-0.05		-0.06–-0.04
**40-50 years**	189 870										
Low education	44 838	(24)	0.05	(0.28)		0.06	(0.65)		0.09	(0.76)	
Low-intermediate	16 709	(9)	0.05	(0.28)		0.04	(0.53)		0.06	(0.60)	
Intermediate	57 799	(30)	0.12	(0.45)		0.04	(0.54)		0.04	(0.50)	
Intermediate-high	56 249	(30)	0.40	(0.99)		0.08	(0.71)		0.03	(0.46)	
High education	14 275	(7)	0.42	(1.12)		0.13	(0.93)		0.02	(0.34)	
Diff (high-low)*			0.37		0.35–0.39	0.07		0.05–0.08	-0.07		-0.08–-0.06
**50-60 years**	175 017										
Low education	37 361	(21)	0.01	(0.14)		0.03	(0.47)		0.23	(1.18)	
Low-intermediate	55 799	(32)	0.01	(0.14)		0.02	(0.33)		0.14	(0.91)	
Intermediate	30 585	(17)	0.05	(0.27)		0.03	(0.42)		0.09	(0.72)	
Intermediate-high	43 086	(25)	0.11	(0.50)		0.05	(0.57)		0.09	(0.75)	
High education	8 186	(5)	0.23	(0.83)		0.08	(0.72)		0.07	(0.64)	
Diff (high-low)*			0.22		0.20–0.24	0.05		0.03–0.06	-0.16		-0.18–-0.14
**60-70 years**	141 300										
Low education	44 228	(31)	0.00	(0.03)		0.02	(0.31)		0.42	(1.57)	
Low-intermediate	56 195	(40)	0.00	(0.05)		0.01	(0.24)		0.30	(1.35)	
Intermediate	13 733	(10)	0.01	(0.08)		0.03	(0.44)		0.27	(1.28)	
Intermediate-high	23 614	(17)	0.01	(0.16)		0.03	(0.41)		0.21	(1.14)	
High education	3 530	(2)	0.04	(0.36)		0.06	(0.64)		0.18	(1.00)	
Diff (high-low)*			0.04		0.03–0.06	0.04		0.03–0.07	-0.25		-0.28–-0.21

**Mean difference between genders and 95% CI based on 1000 bootstrapped samples*

Table S5a. Mean educational differences and 95% CI based on 1000 bootstrapped samples of 10-year state durations for work participation, unemployment, sickness absence, disability pension, economic inactivity, and (early) retirement, for men, stratified by age. See S6b for education, emigration, and death.

	Work (yr)		Unemployed (yr)		Sickness absence (yr)		Disability pension (yr)		Economically inactive (yr)		(Early) retirement (yr)	
**Men**	**mean diff**	**95% CI**	**mean diff**	**95% CI**	**mean diff**	**95% CI**	**mean diff**	**95% CI**	**mean diff**	**95% CI**	**mean diff**	**95% CI**
**30-40 years**												
Ref. Low	5.55		0.83		1.12		1.08		1.07			
Low-intermediate - Low	1.45	1.38–1.53	-0.23	-0.26–-0.21	-0.23	-0.26–-0.19	-0.67	-0.71–-0.62	-0.25	-0.29–-0.22		
Intermediate - Low	2.52	2.47–2.56	-0.50	-0.51–-0.48	-0.55	-0.57–-0.52	-0.96	-1.00–-0.93	-0.39	-0.41–-0.37		
Intermediate-high - Low	2.55	2.51–2.60	-0.58	-0.60–-0.56	-0.79	-0.81–-0.77	-1.04	-1.07–-1.00	-0.37	-0.40–-0.35		
High - Low	2.68	2.63–2.74	-0.68	-0.69–-0.66	-0.97	-0.99–-0.95	-1.07	-1.10–-1.03	-0.41	-0.44–-0.39		
**40-50 years**												
Ref. Low	6.09		0.52		1.04		1.35		0.76			
Low-intermediate - Low	1.20	1.13–1.27	-0.17	-0.18–-0.15	-0.25	-0.28–-0.22	-0.64	-0.69–-0.59	-0.11	-0.14–-0.08		
Intermediate - Low	1.95	1.90–1.99	-0.26	-0.27–-0.25	-0.39	-0.41–-0.38	-1.03	-1.06–-1.00	-0.17	-0.19–-0.15		
Intermediate-high - Low	1.99	1.94–2.04	-0.27	-0.28–-0.25	-0.59	-0.61–-0.57	-1.15	-1.18–-1.12	-0.09	-0.11–-0.07		
High - Low	2.41	2.36–2.46	-0.37	-0.39–-0.36	-0.83	-0.84–-0.81	-1.28	-1.31–-1.25	-0.08	-0.11–-0.06		
**50-60 years**												
Ref. Low	5.13		0.35		0.96		2.66		0.49			
Low-intermediate - Low	1.64	1.58–1.69	-0.10	-0.11–-0.09	-0.16	-0.18–-0.14	-1.21	-1.25–-1.16	-0.03	-0.05–-0.01		
Intermediate - Low	2.32	2.27–2.37	-0.12	-0.13–-0.11	-0.22	-0.24–-0.20	-1.79	-1.83–-1.74	-0.01	-0.03–0.01		
Intermediate-high - Low	2.75	2.70–2.81	-0.15	-0.16–-0.14	-0.46	-0.48–-0.44	-2.11	-2.15–-2.06	0.12	0.10–0.14		
High - Low	3.32	3.27–3.38	-0.24	-0.26–-0.23	-0.67	-0.69–-0.65	-2.43	-2.48–-2.39	0.17	0.14–0.20		
**60-70 years**												
Ref. Low	2.12		0.13		0.38		3.33		0.40		2.89	
Low-intermediate - Low	0.95	0.91–0.99	0.01	0.00–0.02	0.01	0.00–0.02	-1.05	-1.09–-1.00	0.19	0.17–0.21	0.21	0.18–0.24
Intermediate - Low	0.95	0.91–0.99	0.02	0.01–0.03	-0.02	-0.03–-0.01	-1.51	-1.55–-1.46	0.49	0.46–0.52	0.31	0.28–0.34
Intermediate-high - Low	1.81	1.77–1.86	0.00	-0.01–0.01	-0.06	-0.07–-0.05	-2.09	-2.14–-2.05	0.25	0.22–0.27	0.44	0.41–0.48
High - Low	3.07	3.02–3.12	-0.05	-0.06–-0.03	-0.15	-0.16–-0.14	-2.75	-2.79–-2.70	0.32	0.29–0.35	-0.03	-0.06–0.01

Table S5b. Mean educational differences and 95% CI based on 1000 bootstrapped samples of 10-year state durations for men for education, emigration, and death.

	Education (yr)		Emigration (yr)		Death (yr)	
**Men**	**mean diff**	**95% CI**	**mean diff**	**95% CI**	**mean diff**	**95% CI**
**30-40 years**						
Ref. Low	0.09		0.12		0.13	
Low-intermediate - Low	-0.02	-0.02–-0.01	-0.01	-0.02–0.01	-0.05	-0.07–-0.03
Intermediate - Low	0.01	0.01–0.02	-0.05	-0.06–-0.04	-0.09	-0.10–-0.08
Intermediate-high - Low	0.31	0.30–0.32	0.03	0.01–0.04	-0.11	-0.12–-0.10
High - Low	0.44	0.42–0.46	0.13	0.11–0.14	-0.12	-0.13–-0.10
**40-50 years**						
Ref. Low	0.04		0.06		0.15	
Low-intermediate - Low	0.00	-0.01–0.00	0.01	0.00–0.03	-0.05	-0.07–-0.04
Intermediate - Low	0.01	0.01–0.01	-0.02	-0.02–-0.01	-0.09	-0.10–-0.08
Intermediate-high - Low	0.15	0.15–0.16	0.06	0.05–0.06	-0.11	-0.12–-0.10
High - Low	0.12	0.11–0.13	0.14	0.12–0.16	-0.11	-0.12–-0.10
**50-60 years**						
Ref. Low	0.01		0.04		0.35	
Low-intermediate - Low	0.00	0.00–0.00	-0.02	-0.02–-0.01	-0.13	-0.15–-0.11
Intermediate - Low	0.01	0.01–0.01	-0.01	-0.02–0.00	-0.19	-0.20–-0.17
Intermediate-high - Low	0.06	0.05–0.06	0.02	0.01–0.03	-0.23	-0.25–-0.21
High - Low	0.05	0.04–0.06	0.06	0.05–0.07	-0.25	-0.27–-0.23
**60-70 years**						
Ref. Low	0.00		0.02		0.73	
Low-intermediate - Low	0.00	0.00–0.00	-0.01	-0.01–0.00	-0.20	-0.23–-0.18
Intermediate - Low	0.00	0.00–0.00	0.01	0.00–0.02	-0.25	-0.28–-0.23
Intermediate-high - Low	0.00	-0.01–0.01	0.01	0.00–0.02	-0.37	-0.40–-0.35
High - Low	0.01	0.01–0.02	0.02	0.01–0.03	-0.45	-0.48–-0.42

Table S6a. Mean educational differences and 95% CI based on 1000 bootstrapped samples of 10-year state durations for work participation, unemployment, sickness absence, disability pension, economic inactivity, and (early) retirement, for women, stratified by age. See S7b for education, emigration, and death.

	Work (yr)		Unemployed (yr)		Sickness absence (yr)		Disability pension (yr)		Economically inactive (yr)		(Early) retirement (yr)	
**Women**	**mean diff**	**95% CI**	**mean diff**	**95% CI**	**mean diff**	**95% CI**	**mean diff**	**95% CI**	**mean diff**	**95% CI**	**mean diff**	**95% CI**
**30-40 years**												
Ref. Low	4.14		0.85		1.67		1.24		1.75			
Low-intermediate - Low	1.62	1.54–1.70	-0.30	-0.32–-0.27	-0.26	-0.30—0.22	-0.71	-0.76–-0.65	-0.27	-0.32—0.22		
Intermediate - Low	2.39	2.34–2.44	-0.38	-0.40–-0.36	-0.53	-0.56–-0.50	-1.05	-1.09–-1.01	-0.40	-0.43—0.37		
Intermediate-high - Low	2.73	2.68–2.78	-0.60	-0.61–-0.58	-0.82	-0.85—0.79	-1.17	-1.22—1.13	-0.54	-0.57—0.51		
High - Low	2.94	2.89–3.00	-0.64	-0.66–-0.62	-1.14	-1.17—1.11	-1.22	-1.26–-1.18	-0.62	-0.66—0.59		
**40-50 years**												
Ref. Low	5.12		0.50		1.41		1.78		0.99			
Low-intermediate - Low	1.40	1.33–1.47	-0.20	-0.21–-0.18	-0.22	-0.25–-0.18	-0.82	-0.88–-0.77	-0.11	-0.15—0.08		
Intermediate - Low	1.93	1.88–1.97	-0.18	-0.20–-0.17	-0.38	-0.40—0.36	-1.23	-1.27–-1.20	-0.12	-0.15—0.10		
Intermediate-high - Low	2.18	2.14–2.23	-0.29	-0.30–-0.28	-0.55	-0.57—0.53	-1.45	-1.49–-1.42	-0.20	-0.22—0.17		
High - Low	2.69	2.63–2.74	-0.32	-0.34–-0.31	-0.86	-0.89—0.84	-1.63	-1.67—1.59	-0.24	-0.27—0.21		
**50-60 years**												
Ref. Low	4.11		0.29		1.07		3.61		0.65			
Low-intermediate - Low	1.79	1.74–1.84	-0.08	-0.09–-0.07	-0.09	-0.11—0.07	-1.43	-1.48–-1.38	-0.08	-0.10—0.06		
Intermediate - Low	2.61	2.55–2.66	-0.07	-0.08–-0.06	-0.06	-0.09—0.04	-2.37	-2.42–-2.31	0.00	-0.03–0.02		
Intermediate-high - Low	3.12	3.07–3.17	-0.15	-0.16–-0.14	-0.22	-0.24—0.20	-2.67	-2.72–-2.62	-0.06	-0.09—0.04		
High - Low	3.59	3.51–3.66	-0.14	-0.15–-0.12	-0.44	-0.47—0.42	-3.13	-3.19–-3.07	0.02	-0.02–0.06		
**60-70 years**												
Ref. Low	1.53		0.09		0.29		3.90		0.98		2.87	
Low-intermediate - Low	0.90	0.87–0.93	0.04	0.03–0.05	0.05	0.05–0.06	-1.07	-1.11–-1.03	-0.08	-0.11—0.05	0.29	0.26–0.31
Intermediate - Low	1.52	1.47–1.57	0.04	0.03–0.05	0.10	0.09–0.11	-1.77	-1.83–-1.72	-0.05	-0.09—0.01	0.29	0.26–0.33
Intermediate-high - Low	1.76	1.73–1.81	-0.02	-0.03–-0.01	0.10	0.09–0.11	-1.92	-1.98–-1.87	-0.34	-0.37—0.31	0.61	0.58–0.64
High - Low	3.08	2.98–3.17	-0.01	-0.02–0.01	0.09	0.06–0.11	-2.80	-2.89–-2.71	-0.37	-0.42—0.31	0.17	0.10–0.23

Table S6b. Mean educational differences and 95% CI based on 1000 bootstrapped samples of 10-year state durations for women for education, emigration, and death.

	Education (yr)		Emigration (yr)		Death (yr)	
**Women**	**mean diff**	**95% CI**	**mean diff**	**95% CI**	**mean diff**	**95% CI**
**30-40 years**						
Ref. Low	0.15		0.14		0.05	
Low-intermediate - Low	-0.05	-0.06–-0.04	-0.01	-0.03–0.01	-0.03	-0.04–-0.02
Intermediate - Low	0.06	0.05–0.06	-0.05	-0.06–-0.04	-0.04	-0.04–-0.03
Intermediate-high - Low	0.42	0.41–0.43	0.01	-0.01–0.02	-0.04	-0.05–-0.03
High - Low	0.62	0.60–0.64	0.11	0.09–0.13	-0.05	-0.06–-0.04
**40-50 years**						
Ref. Low	0.05		0.06		0.09	
Low-intermediate - Low	-0.01	-0.01–0.00	-0.02	-0.03–-0.01	-0.03	-0.04–-0.02
Intermediate - Low	0.06	0.06–0.07	-0.02	-0.03–-0.01	-0.05	-0.06–-0.04
Intermediate-high - Low	0.35	0.34–0.36	0.01	0.01–0.02	-0.06	-0.07–-0.05
High - Low	0.37	0.35–0.39	0.07	0.05–0.08	-0.07	-0.08–-0.06
**50-60 years**						
Ref. Low	0.01		0.03		0.23	
Low-intermediate - Low	0.00	0.00–0.00	-0.02	-0.02–-0.01	-0.09	-0.11–-0.08
Intermediate - Low	0.03	0.03–0.04	-0.01	-0.01–0.00	-0.14	-0.15–-0.13
Intermediate-high - Low	0.10	0.09–0.10	0.02	0.01–0.02	-0.14	-0.15–-0.12
High - Low	0.22	0.20–0.24	0.05	0.03–0.06	-0.16	-0.18–-0.14
**60-70 years**						
Ref. Low	0.00		0.02		0.42	
Low-intermediate - Low	0.00	0.00–0.00	-0.01	-0.01–0.00	-0.12	-0.14–-0.10
Intermediate - Low	0.01	0.00–0.01	0.02	0.01–0.02	-0.15	-0.18–-0.12
Intermediate-high - Low	-0.02	-0.03–-0.01	0.01	0.01–0.02	-0.21	-0.23–-0.19
High - Low	0.04	0.03–0.06	0.05	0.03–0.07	-0.25	-0.28–-0.21
